# Identification and phylogenetic analysis of marine sponges in the Jordanian Gulf of Aqaba using DNA barcoding

**DOI:** 10.1016/j.heliyon.2025.e42771

**Published:** 2025-02-18

**Authors:** Zeinab Arabeyyat, Mais Sweiss, Abdalmajeed Alajlouni, Neda'a Al-Ajlouni, Marwan Mahmoud, Sura Shartooh, Farah Alsoqi, Maysoon Kteifan

**Affiliations:** a Department of Marine Biology, The University of Jordan, Aqaba, 77110, Jordan; b Department of Biotechnology, Al-Balqa Applied University, Al-Salt, Jordan; c Veterinary Pathology and Public Health Department, JUST, Irbid, Jordan; d ASEZA/ BEN-Hayyan Labs, Aqaba, 77110, Jordan; e Aqaba Marine Reserve, Aqaba, Jordan

**Keywords:** Porifera, DNA barcode, Phylogeny, rRNA genes, Taxonomy, Red Sea

## Abstract

Sponges (Porifera) are the largest biomass component of coral reefs benthic fauna among marine organisms and are very morphologically diverse. In the present work, we aimed to identify marine sponges in the Jordanian Gulf of Aqaba using the partial 18S rRNA and the 28S rRNA genes as DNA barcoding markers. A total of nine morphologically different marine sponge samples from 6.6m to approximately 22m depth were collected. Sponge fragments were frozen at −80 °C prior to DNA extraction. The sponge's DNA was extracted using a commercial kit and subjected directly to PCR amplification for the 18S rRNA and 28S rRNA genes. The DNA sequences were analyzed using the Basic Local Alignment Search Tool (BLAST) to determine the sponge's identity, and phylogenetic trees were constructed to clarify the relationship among the samples. The results obtained revealed the presence of the following genera: *Axinella*, *Negombata*, *Siphonochalina*, *Diacarnus*, and an unidentified genus within the order *Haplosclerida*. Identification of sponge species was difficult due to the scarcity of diagnostic morphological characters. To our knowledge, this is the first study in the Jordanian Gulf of Aqaba that focuses on the morphological and molecular taxonomy of marine sponges using DNA barcoding markers.

## Highlights

• The marine sponges of the Gulf of Aqaba, Jordan, are still unknown.
• New protocol to identify different types of sponges at the genus level.
• Genera found: *Axinella*, *Negombata*, *Siphonochalina*, *Diacarnus* and an unidentified genus within the order *Haplosclerida.*
• The phylogenetic approach can achieve a level of confidence for sponge identification.
• This is the first morpho-molecular identification of Porifera in the Gulf of Aqaba.

## 1 Introduction

Coral reefs are important habitats with the most diverse of all marine ecosystems. They provide a home for at least a fourth part of marine fauna, including sponges, mollusks, echinoderms, fishes, cnidarians, crustaceans, and others [1]. Coral reefs are known worldwide as fragile ecosystems that are exposed to many threats such as climate change, ocean acidification, illegal fishing, and harmful land use practices [2]. The coral reefs in the northern Red Sea are also vulnerable to human impacts such as tourism, shipping, pollution, and port activities [3].

One of the largest biomass components of coral reefs benthic fauna is sponges in the Caribbean sponges [4]. While sponges (Phylum Porifera) are essential for the functioning of the coral reef ecosystem [5], relatively little is known about sponge species diversity in the Red Sea [6]. Our current knowledge of the Red Sea sponge is based on Wooster and his colleagues [7] and Erpenbeck and his colleagues [8,9], where it harbors a diverse understudied community of sponge species with around 261 valid sponge species (representing 114 genera) in the Red Sea [7]. However, the Jordanian Gulf of Aqaba is still considered an underexplored Gulf region. This gulf is part of a semi-enclosed basin that is separated from the Red Sea at the Straits of Tiran, spans 27 km in length, and is known as one of the unique aquatic ecosystems that has one of the world's richest coral communities [10].

The Red Sea is known as a high biodiversity and endemism region [7]. However, little is still known about the biodiversity of Red Sea sponges [8], especially regarding data on their abundance, coverage, and species composition that remain scarce [6]. This limitation in data is due to limited taxonomic knowledge, the cryptic growth forms of sponges, and the challenges of identifying them in the field [11].

Morphological identification of sponges based only on spicule identification is a difficult task, as the morphology of sponge spicules can be simple, complex, or even absent [12]. Therefore, genetic analysis of sponges can shed light on their unique evolution, and sponges may prove to be ideal model organisms for addressing questions of evolutionary and ecological genetics [6]. Furthermore, molecular tools enable the assessment of phylogenetic relationships in sponges, ranging from intra-species to phylum-level relationships [8], despite the absence of a distinct barcoding gap and the lack of a genetic distance threshold-based species concept for sponges [8,13].

Marine sponges have unique phylogenetic relationships and genetic diversity that can be effectively explored through the combined use of morphological analysis and molecular barcoding. In the present study we therefore assessed the morphological and molecular identification of sponges using two nuclear ribosomal rRNA genes, the large subunit 28S rRNA and the small subunit 18S rRNA. These two markers were utilized for molecular barcoding and phylogenetic analysis. Additionally, to our knowledge, no prior study has focused on clarifying sponge with morphological and molecular identification of sponges in the Jordanian Gulf of Aqaba.

## 2 Materials and methods

### 2.1 Sponge samples collection

**Fig. 1:**
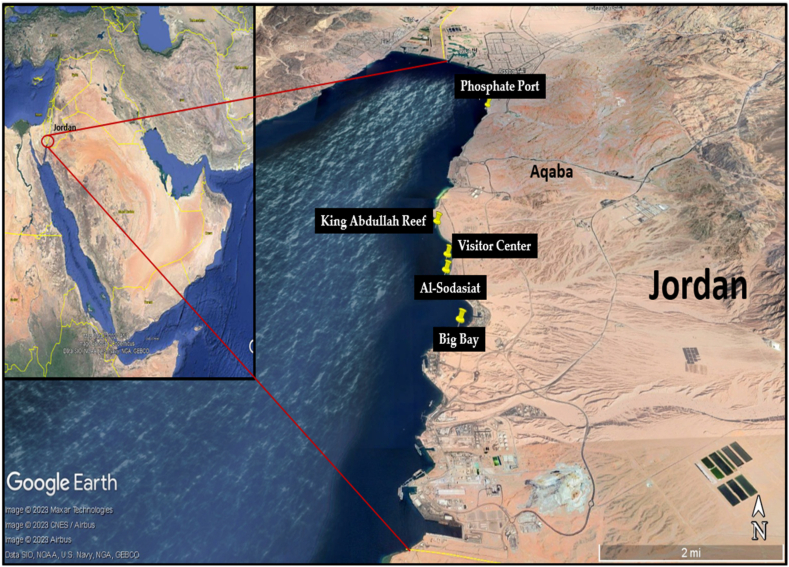
Collection sites of samples used in this study. Map created with Google Earth Pro (Google LLC., Mountain View, CA, USA).

Sponge samples were collected from an inshore area in Jordan's marine environment by SCUBA diving. The Jordanian Gulf of Aqaba is known for its small coral reef ecosystems that cover only 4 km^2^ in total along the 27 km of the entire coastline. Nine sponge samples were collected from five inshore reefs (Fig. 1) along the short coast of the Jordanian Gulf of Aqaba (29°31′36.01"N 35°00′28.01"E). Fragments of sponge individuals were collected at a depth range of 6.6–22 m. The geographic coordinates of the five sampling sites are shown in Table 1. Sponges were collected during December 2022. The temperature of the water was around 25 °C, and all sponges were emergent species living in an area that was exposed to light. Sponge fragments were collected and placed separately into a sterile plastic bag; the bags were then stored on ice for transport to the laboratory. In the laboratory, all samples were immediately placed at −80 °C prior to further analysis.

**Table 1:** Geographic coordinates and depth of sponge sampling sites.

Sampling site	Sample IDs	Depth (m)	Coordinates
Big Bay	SP1	17.8	29°24′18.97"N/34°58′23.79"E
	SP2	18	
	SP3	13.3	
Visitor Center	SP4	6.6	29°25′36.41"N/34°58′17.14"E
Phosphate Port	SP5	16.4	29°30′9.48"N/34°59′30.81"E
King Abdullah Reef	SP6	16.5	29°26′23.34"N/34°58′8.80"E
	SP7	16.7	
	SP8	22	
Al-Sodasiat	SP9	9.5	29°25′16.42"N/34°58′14.45"E

### 2.2 Morphology of sponge samples

The morphological identification methods using the external morphology and spicules of sponges were used to identify collected sponges in this study based on the descriptions of various sponges from different regions available in the literature [7,[14], [15], [16], [17]]. Each sponge species has one or more types of spicules, each with a specific morphology [12]. In the present study, eight sponge samples out of the original nine samples were identified by determining the structure of their spicules (SP7 is not included here, but was tested for molecular identification). Spicule analysis was carried out using the technique described in Hooper (2000) using a rapid tissue digestion in bleach [18]. Sponge fragments of approximately 5–10 mm were placed in 15 ml conical tubes and digested with 5 ml active bleach (Spartan©, Jordan) and incubated until all tissue dissolved. Bleach was diluted with water and then replaced with ethanol. A clean spicule suspension was aspirated and pipetted onto a microscope slide. Slides were analyzed under magnifications of 50–400x on an inverted microscope (AX10 Vert.A1, Zeiss, Germany).

### 2.3 Total genomic DNA extraction and PCR amplification

Total genomic DNA of nine sponge samples was extracted using the G-spin™ Genomic DNA Extraction Kit (iNtRON Biotechnology, South Korea) following the manufacturer's protocol. The quality of the DNA extracts was determined by 1.5 % agarose gel electrophoresis. The 28S rRNA partial gene was amplified using a sponge universal forward primer C1’ (5′-ACCCGCTGAATTTAAGCAT-3′), and reverse primer D2’ (5′-TCCGTGTTTCAAGACGGG-3′) [19]. Thermocycler conditions for the C1′-D2′ primer pair were as follows: the first cycle is 94 °C for 4 min, annealing at 57 °C for 2 min, and extension at 72 °C for 2 min, followed by 30 cycles, each consisting of 1 min at 94 °C, 1 min at the annealing temperature, and 1 min at 72 °C. Followed by a final extension step of 4 min at 72 °C [19]. The 18S rRNA gene fragment was amplified using the forward primer SS5 (5′-GGTTGATCCTGCCAGTAGTCATATGCTTG-3′), and reverse primer SS3 (5′-GATCCTTCCGCAGGTTCACCTACGGAAACC-3′) [20]. The thermocycler conditions for the 18S-rRNA primers were as follows: the first cycle is 94 °C for 2:30 min, annealing at 56 °C for 1 min, and extension at 72 °C for 2 min, followed by 29 cycles, each consisting of 1 min at 94 °C, annealing at 56 °C for 1 min, and 2:30 min at 72 °C. Followed by a final extension step of 4 min at 72 °C ended the reaction [21]. PCR products were analyzed on 1 % agarose gels using RedSafe™ nucleic acid staining solution (iNtRON Biotechnology, South Korea). The expected PCR product sizes were 800–900 bp for 28S rRNA (C1’ – D2′ primers) and ∼1800 bp for 18S rRNA with (SS5 – SS3 primers).

### 2.4 Sequencing and phylogenetic analysis of amplified PCR products

Direct sequencing of the PCR products for the 18S and 28S rRNA genes of the sponge was conducted using the Sanger sequencing service from Macrogen (Macrogen Inc., Korea). Sequences were edited by removing low-quality sequences at the beginning and the end of the chromatogram. Edited sequences were used for similarity search using the BLASTn tool at the National Center for Biotechnology Information (NCBI) [22]. GenBank accession numbers for sponges (n = 9) collected in this study were submitted to GenBank (NCBI) and have the following accession numbers: 18S rRNA (accession no. OR532378–OR532386) and 28S rRNA (accession no. OR532387–OR532395). To clarify the phylogenetic relationship among the collected samples and the sponge genera in GenBank, sequences were aligned using the ClustalW algorithm, with a sequence length after trimming of 991 nt for the 18S rRNA and 735 nt for the 28S rRNA. The bootstrapping value was 10000 replicates. The maximum likelihood phylogenetic tree was constructed based on the Tamura-Nei model [23] using MEGA X [24]. *Phycopsis* sp. (KC902035.1) and *Desmanthus incrustans* (HQ379195.1) were outgroups for the 18S rRNA and 28S rRNA phylogenetic trees, respectively.

## 3 Results

### 3.1 Identification of sponges

Based on the external characters [14], SP1 grows as a massive sponge; SP2 is like an erect and branched sponge; SP3 includes cups and thin cylindrical branches morphologies; SP4 is like a composite massive sponge; SP5 has cup- and barrel-shaped forms; SP6 is like a thick, erect, branched sponge; SP7 has a thick, erect, and rounded branch; SP8 has a massively encrusting shape. While SP9 has a flabellate shape.

**Fig. 2:**
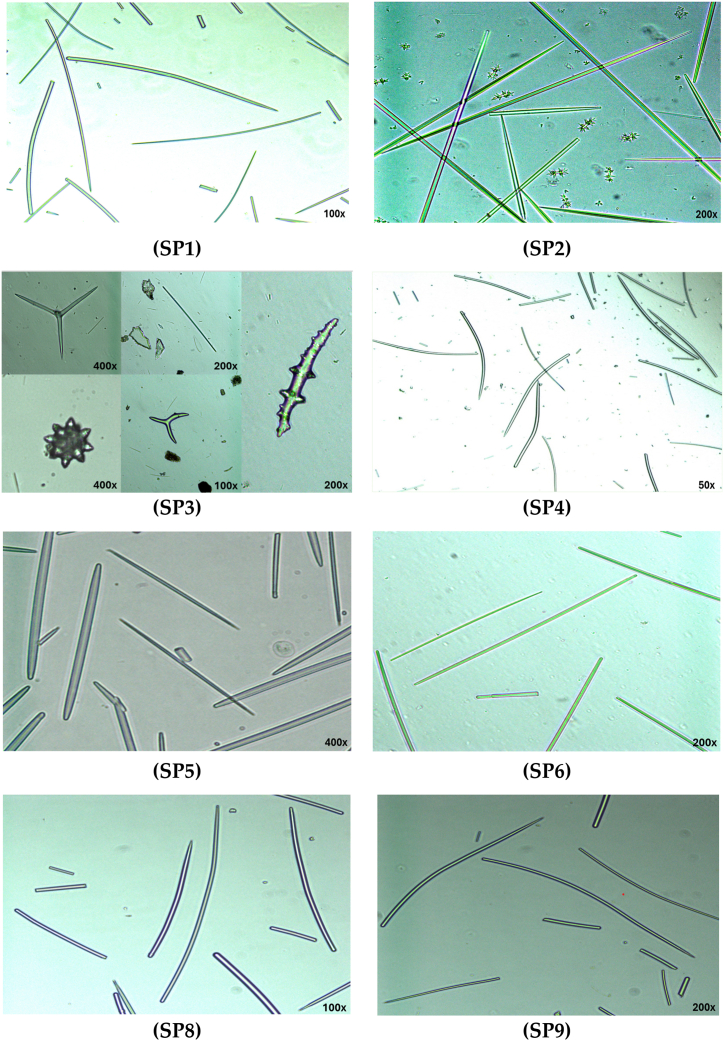
Representative spicules of sponge samples SP1 – SP9. SP7 was not available in this analysis.

For the morphologies of spicules, only eight sponge spicule samples were analyzed for sponge identification using the inverted microscope (only sample SP7 was missing when analyzed). Examples of the spicule morphology of the preparations analyzed are presented in Fig. 2. The shape and sizes of spicules in each sponge are variable. Based on the presence of only style spicules [16], we concluded that samples SP1, SP4, and SP9 belong to the same genus, *Axinella*. The spicules from sample SP2 are oxea, strongyle, and sanidaster spicules that match the description of *Negombata magnifica* [25]. Spicules from sample SP3 are spheroxyasters, acanthoxea spicules, styles, and tripod spicules. While spicules from sample SP5 are oxea and strongyle spicules from the *Siphonochalina* genus [26], spicules from sample SP6 are oxea spicules of the *Diacarnus* genus [25], and spicules from sample SP8 were only style spicules with one end pointed and the other rounded. Based only on morphological characters, it was not possible to determine the genera for samples SP3 and SP8.

**Fig. 3:**
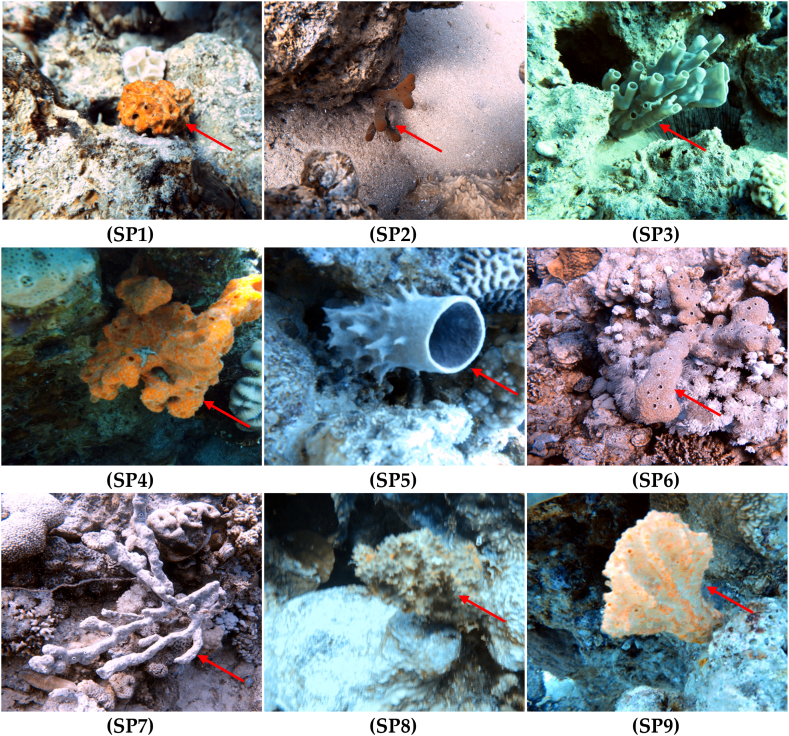
Underwater photographs of all sponges sampled in the present study. Sponges photographs were collected by scuba divers. These photographs have been color corrected using an algorithm developed by Nikolaj Bech Andersen [31].

For molecular identification, a total of nine 18S and nine 28S sequences were successfully obtained from nine samples (see Fig. 3 for sponge samples). The partial nuclear 18S and 28S rRNA sequences have been analyzed and NCBI BLASTN [22] was used to find similar sequences.

The novelty of this study is that it is the first morphological and molecular identification of marine sponges in the Jordanian Gulf of Aqaba, which has yet to be studied much by previous researchers. In the current study, the morphology characteristics support results of the molecular analysis and match those of SP1 (*Axinella* sp.), SP2 (*Negombata magnifica*), SP4 (*Axinella* sp.), SP5 (*Siphonochalina* sp.), SP6 (*Diacarnus* sp.), and SP9 (*Axinella* sp.) [[27], [28], [29], [30]]. However, it was difficult to identify the collected sponges to the species level, except for SP2.

Based on the sequencing analysis, all sequences from sponge samples were identified and classified as belonging to the following five genera: *Axinella*, *Negombata*, *Siphonochalina*, *Diacarnus*, and an unidentified genus within the order *Haplosclerida*, with identity percentages ranging from 98.29 to 100. The genus *Axinella* was predominant in 4 out of 5 sampling sites. Identification at the species level was possible for only one sample (SP2). The samples were identified at the genus level because they had identical sequences (the markers did not vary), and it was not possible to determine the species. For SP8, the highest identity based on the 28S rRNA sequence was with *Haliclona oculata* HQ379251.1 and *Callyspongia* sp. 1 JV-2020 MW016057.1; but the low identity (around 86 %) suggests that SP8 does not belong to these genera. However, the highest identity based on the 18S RNA was around 98.29 % for the *Cladocroce* sp. 0CDN9562-C and *Calyx* sp. NIWAKD1132, supporting its classification within these genera. Therefore, we recommend classifying SP8 within a genus of the order *Haplosclerida*. Additionally, some sequences of the 28S rRNA gene have very low identity with sequences available on GenBank (such as SP3, SP5, and SP8), and that could be due to the lack of information in the GenBank database. At 86.63 % identity, SP3 has the lowest identity for the 18S rRNA sequences (Table 2).

**Table 2:** BLAST analysis of PCR amplicons from marine sponge isolates according the highest similarity to 18S and 28S rRNA sequences in the GenBank nucleotide sequence database.

Sponge IDs	Locus	Size (nt)	Closet organism in GenBank & NCBI accession no.	Max score	Query (%)	Identity (%)	E-value	GenBank accession no.^b^
SP1	28S	700	a*Axinella* sp. (AY561925.2)	1242	100	100	0.00	OR532387
			a*Axinella corrugate* (AY864741.1)	1214	100	99	0.00	
	18S	950	a*Axinella verrucosa* (KX622144.1)	1620	100	99.11	0.00	OR532378
			*Axinella corrugate* (KC901907)	1620	100	99.11	0.00	
			a*Axinella corrugate* (KC901878.1)	1615	100	99	0.00	
			a*Prosuberites laughlini* (MZ416700.1)	1615	100	99	0.00	
SP2	28S	763	a*Negombata magnifica* (OM406238.1)	994	100	100	0.00	OR532388
			*Negombata* sp. (OM406250.1)	983	100	99.63	0.00	
	18S	1050	a*Negombata magnifica* (FR819687.1)	1936	100	99.90	0.00	OR532379
			*Neopodospongia cf*. *normani* (KC902112.1)	1886	100	99.05	0.00	
			*Diacarnus* sp. (OP895635.1)	1845	100	98.38	0.00	
SP3	28S	700	a*Callyspongia* sp. (MW016057.1)	767	99	86.63	0.00	OR532389
			*Haplosclerida* sp. (MW016166.1)	761	99	86.63	0.00	
			a*Haliclona oculate* (HQ379251.1)	728	100	85.82	0.00	
	18S	1000	a*Siphonochalina* sp. (DQ927311.1)	1814	100	99.4	0.00	OR532380
			a*Cladocroce* sp. (KC902202.1)^a^	1751	100	98.3	0.00	
			*Calyx* sp. (DQ927313.1)	1751	100	98.3	0.00	
SP4	28S	722	a*Axinella* sp. (AY561925.2)	1251	100	100	0.00	OR532390
			a*Axinella corrugate* (AY864741.1)	1223	100	99.26	0.00	
	18S	1000	a*Axinella verrucosa* (KX622144.1)	1803	100	99.2	0.00	OR532381
			*Axinella corrugate* (KC901907.1)	1803	100	99.1	0.00	
			a*Prosuberites laughlini* (EF654529.1)	1797	100	99.1	0.00	
SP5	28S	800	a*Callyspongia* sp. (MW016057.1)	761	97	86.09	0.00	OR532391
			*Haplosclerida* sp. (MW016166.1)	754	97	85.95	0.00	
			a*Haliclona oculata* (HQ379251.1)	750	97	86.07	0.00	
	18S	1000	a*Siphonochalina* sp. (DQ927311.1)	1814	100	99.4	0.00	OR532382
			a*Cladocroce* sp. (KC902202.1)	1751	100	98.3	0.00	
			*Calyx* sp. (DQ927313.1)	1751	100	98.3	0.00	
SP6	28S	764	a*Diacarnus* sp. (KU060338.1)	737	55	99.75	0.00	OR532392
			*Negombata* sp. (OM406250.1)	769	85	89.68	0.00	
	18S	1000	a*Diacarnus* sp. (OP895635.1)	1847	100	100	0.00	OR532383
			*Diacarnus bismarckensis* (KC902137.1)	1836	100	99.8	0.00	
			*Neopodospongia cf*. *normani* (KC902112.1)	1777	100	98.7	0.00	
SP7	28S	709	a*Axinella* sp. (AY561925.2)	1243	100	100	0.00	OR532393
			a*Axinella corrugate* (AY864741.1)	1216	100	99.26	0.00	
	18S	1000	a*Axinella verrucosa* (KX622144.1)	1814	100	99.4	0.00	OR532384
			*Axinella corrugate* (KC901907.1)	1814	100	99.4	0.00	
			a*Prosuberites laughlini* (EF654529.1)	1808	100	99.3	0.00	
SP8	28S	750	a*Haliclona oculata* (HQ379251.1)	811	97	87.62	0.00	OR532394
			*Haliclona melissae* (OP526584.1)	791	95	87.59	0.00	
			a*Callyspongia* sp. (MW016057.1)	760	97	86.15	0.00	
	18S	1050	a*Cladocroce* sp. (0CDN9562-C) (KC902202.1)	1836	100	98.29	0.00	OR532385
			*Calyx* sp. (DQ927313.1)	1836	100	98.29	0.00	
			*Cladocroce* sp. (KT900335.1)	1831	100	98.19	0.00	
SP9	28S	700	a*Axinella* sp. (AY561925.2)	1234	100	100	0.00	OR532395
			a*Axinella corrugate* (AY864741.1)	1206	100	99.25	0.00	
	18S	900	a*Axinella verrucosa* (KX622144.1)	1640	100	99.56	0.00	OR532386
			*Axinella corrugate* (KC901907.1)	1640	100	99.56	0.00	
			a*Prosuberites laughlini* (EF654529.1)	1635	100	99.44	0.00	

Please note that before doing BLAST, all the sequences were trimmed from the beginning and the end to remove the bad quality and low signal sequences.

a These sequences are the reference sequences used in constructing the phylogenetic tree.

b GenBank accession number of all samples used in this study.

### 3.2 Phylogenetic analysis

**Fig. 4:**
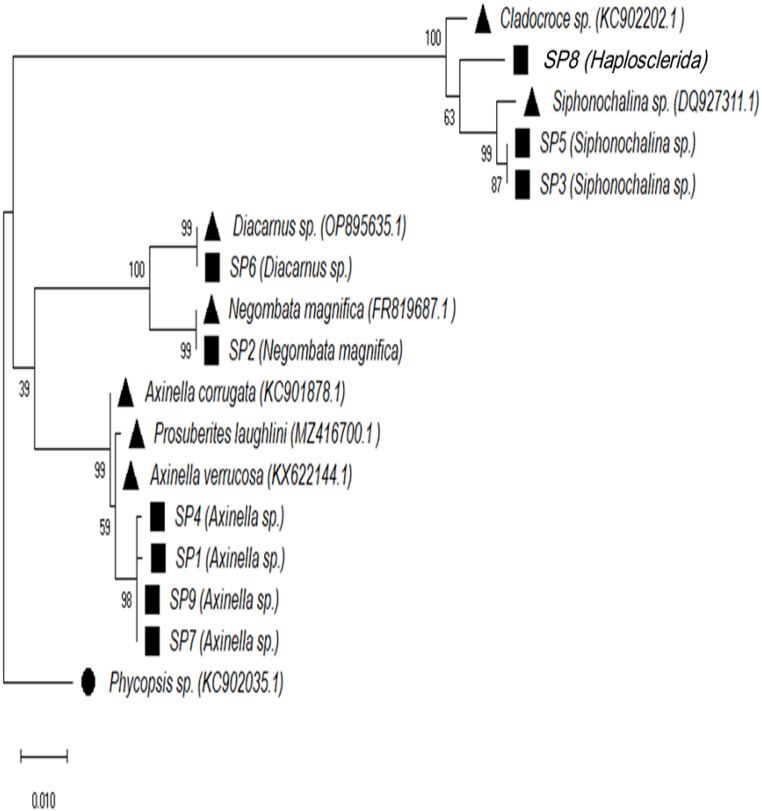
The maximum likelihood phylogenetic tree of the 18S rRNA gene. The tree was constructed using Maximum Likelihood method based on the Tamura-Nei model [23]. The boot-strapping value was 10000 and the number next to the branch indicates the percentage of trees in which the associated taxa clustered together. The scale bar is the scale to which the tree was drawn to. Evolutionary analyses were conducted in MEGA X [24]. Black circle, outgroup to which the tree was rooted to; black triangles; representative sequences from the GenBank and their accession numbers; black squares, the collected samples.

**Fig. 5:**
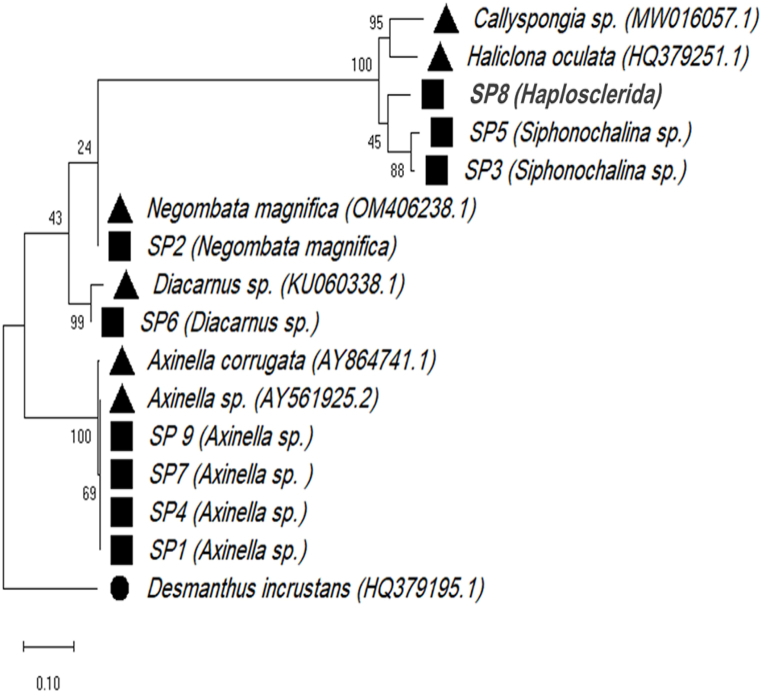
The maximum likelihood phylogenetic tree of the 28S rRNA gene. The tree was constructed using Maximum Likelihood method based on the Tamura-Nei model [23]. The boot-strapping value was 10000 and the number next to the branch indicates the percentage of trees in which the associated taxa clustered together. The scale bar is the scale to which the tree was drawn to. Evolutionary analyses were conducted in MEGA X [24]. Black circle, outgroup to which the tree was rooted to; black triangles; representative sequences from the GenBank and their accession numbers; black squares, the collected samples.

Two maximum likelihood phylogenetic trees were constructed for the collected samples (SP1–SP9) as well as representatives of related genera from GenBank. The phylogenetic trees were constructed based on 18S rRNA (Fig. 4) and 28S rRNA (Fig. 5). The phylogenetic analysis of all isolates from each cluster revealed that there were at least five different genera of marine sponges (*Axinella*, *Negombata*, *Siphonochalina*, *Diacarnus*, and an unidentified genus within the order *Haplosclerida*).

## 4 Discussion

Little information is available about Red Sea sponge [6,7], while no official data were found for the Jordanian Gulf of Aqaba sponges [7]. To our knowledge, no prior study has focused on clarifying sponge with morphological and molecular taxonomy of marine sponges in the Jordanian Gulf of Aqaba. The results obtained, using partial 18S rRNA and the 28S rRNA genes as DNA barcoding markers, revealed the presence of genera like *Axinella*, *Negombata*, *Siphonochalina*, *Diacarnus*, and a genus from the order *Haplosclerida*.

Identification of sponge species is difficult due to the scarcity of diagnostic morphological characters [32]. There are many sponges with little or no spicule diversity, and some sponge families showed a high degree of similarity in their spicules [33]. Therefore, this would preclude discrimination of closely related sponge species. In the current study, we conducted both morphological and molecular methods to identify the collected sponge samples. Based only on spicule type, it was not possible to identify SP1, SP2, SP3, SP4, SP6, SP7, SP8, and SP9. The sample SP5 was similar to the description of genus *Siphonochalina* [29,34] based on external morphology.

DNA barcoding is a powerful tool for sponge identification, although, according to literature studies about Poriferan DNA markers, there is no single ideal marker for all sponge species [[35], [36], [37], [38]]. While morphological identification of sponges is impossible with only loose identification, DNA fragments can be used for targeting DNA barcodes such as 18S rDNA, 28S rDNA, rDNA ITS, and fragment mtDNA cytochrome oxidase subunit 1 (CO1) [32]. The incomplete sequence entries in GenBank are also limiting sponge species identification besides the potential for misidentification [39]. Study of the molecular biodiversity of demosponges of the Red Sea using a partial sequence of the 28S rRNA and mtDNA, the standard animal barcoding fragment revealed that 28S sequences were more variable in Order *Haplosclerida* and can be useful to discriminate the OTUs in order to detect the genetic variation in specimens with identical genotypes for COI mtDNA [8]. Opposing this data, in the present study, the 18S rRNA gene was shown to be more variable than the 28S rRNA.

Two DNA markers were used in this study to identify the collected sponge samples at the molecular level. The 18S rRNA helps to identify sponge samples to genus level for SP3, SP5, and SP8 samples compared to the 28S rRNA results. This could be due to the sequences submitted in the database for the 28S being limited and not covering all the genera of sponges, while the 18S rRNA may cover those sponge genera.

The genus *Axinella* was detected in four out of five sampling sites in this study. Genus *Axinella* (order: *Halichondrida*; family: *Axinellidae*) was found and reported as endemic in the Red Sea [8,40]. The sponge *Negombata magnifica* was detected in single samples collected in the Big Bay site. This toxic finger sponge, *N. magnifica*, was reported as an endemic species in the Red Sea under the previous name *Latrunculia magnifica* [40]. *N. magnifica* was also reported in the Red Sea, Eilat in 2012 by Belinky and his colleagues in 2012 [41]. Moreover, the sponge *Siphonochalina* sp. was detected in two samples from two different sites (Phosphate Port and Big Bay sites). Genus *Siphonochalina* was among the subjects of secondary metabolite research in the Red Sea [42]. While *Callyspongia* (*Callyspongia*) *siphonella*, *Callyspongia* (*Callyspongia*) *tubulosa*, *Callyspongia* (*Euplacella*) communis, and *Callyspongia reticulata* were reported from the Red Sea under the following previous names: *Siphonochalina siphonella*, *Siphonochalina tubulosa*, *Siphonochalina communis*, and *Siphonochalina reticulata*, respectively [40,43,44].

In this study, the *Diacarnus* genus was detected in single samples in the King Abdullah Reef site. The distribution of the genus *Diacarnus* (order: *Poecilosclerida*; family: *Podospongiidae*) includes the Red Sea [30]. *Diacarnus erythraeanus* and *Diacarnus spinipoculum* were also reported and studied for secondary metabolites in the Red Sea [[45], [46], [47], [48], [49]]. While a marine sponge from an unidentified genus within the order *Haplosclerida* was detected in the SP8 sample from Al-Sodasiat site in this study. Notably, our results showed that the highest identity based on the 28S rRNA sequence was with *Haliclona oculata* HQ379251.1 and *Callyspongia* sp. 1 JV-2020 MW016057. Meanwhile, the highest identity based on the 18S RNA (around 98.29 %) was with the *Cladocroce* sp. 0CDN9562-C and *Calyx* sp. NIWAKD1132. These results suggest that the genus of this sample could not be determined based on the available data. Therefore, in this study, we classify it as an unidentified genus within the order *Haplosclerida*. Additionally, the lowest identity values were observed for the sequences of SP3 (86.63 %), SP5 (87.05 %), and SP8 (86.6 %), respectively (Table 2), and this could be due to the lack of information in the GenBank database. The results of phylogenetic analysis based on the 18S and 28S rRNA gene sequences imply recognition of five genera (*Axinella*, *Negombata*, *Siphonochalina*, *Diacarnus*, and an unidentified genus within the order *Haplosclerida*). It is important for sponge identification to consider not only spicules and the outer morphology but also to apply histological techniques and molecular analysis with multiple DNA markers. Additionally, analyzing spicule morphology in more detail and expanding molecular barcoding with additional markers could provide a more comprehensive understanding of the order-level richness and diversity of sponges in the Red Sea region.

## 5 Conclusions

This is the first investigation of morphological and molecular identification of marine sponges in the Jordanian Gulf of Aqaba. Giving consideration to the limitations of each of the molecular and morphological approaches, examination of both of the morphological features and the molecular identification is essential to achieve a more reliable identity [39]. Also, the phylogenetic approach will further enrich our understanding of sponge diversity in the region. However, according to previous studies, knowledge of sponge taxonomy in the Red Sea remains in early stages; hence, more research is needed to fully document the sponge species present in the Red Sea. Upgraded databases are urgently needed as guides for future work.

## CRediT authorship contribution statement

**Zeinab Arabeyyat:** Writing – review & editing, Writing – original draft, Visualization, Validation, Supervision, Software, Resources, Project administration, Methodology, Investigation, Funding acquisition, Formal analysis, Data curation, Conceptualization. **Mais Sweiss:** Writing – review & editing, Validation, Software, Resources, Methodology, Investigation, Formal analysis, Data curation, Conceptualization. **Abdalmajeed Alajlouni:** Methodology, Conceptualization. **Neda'a Al-Ajlouni:** Methodology. **Marwan Mahmoud:** Methodology. **Sura Shartooh:** Methodology. **Farah Alsoqi:** Software, Methodology. **Maysoon Kteifan:** Software, Methodology.

## Ethical statement

No ethical statement was reported.

## Data availability statement

Data will be made available on request.

## Funding

This research received no specific grant from any funding agency in the public, commercial, or not-for-profit sectors.

## Declaration of competing interest

The authors declare that they have no known competing financial interests or personal relationships that could have appeared to influence the work reported in this paper.

## References

[1] El-Naggar H.A. (2021).

[2] Wilkinson S., Brodie J.E.Jone., Frew S. (2011).

[3] Kochzius M. (2007). Community structure of coral reef fishes in El Quadim Bay (El Quseir, Egyptian Red Sea coast). Zool. Middle East.

[4] Wulff J. (2012). Ecological interactions and the distribution, abundance, and diversity of sponges. Adv. Mar. Biol..

[5] Wilkinson C.R. (1983). Net primary productivity in coral reef sponges. Science.

[6] Berumen M.L., Hoey A.S., Bass W.H., Bouwmeester J., Catania D., Cochran J.E., Khalil M.T., Miyake S., Mughal M.R., Spät J.L., Saenz-Agudelo P. (2013). The status of coral reef ecology research in the Red Sea. Coral Reefs.

[7] Wooster M.K., Voigt O., Erpenbeck D., Wörheide G., Berumen M.L., Voolstra C., Berumen M. (2019).

[8] Erpenbeck D., Voigt O., Al-Aidaroos A.M., Berumen M.L., Büttner G., Catania D., Guirguis A.N., Paulay G., Schätzle S., Wörheide G. (2016). Molecular biodiversity of Red Sea demosponges. Mar. Pollut. Bull..

[9] Erpenbeck D., Gholami A., Hesni M.A., Ranjbar M.S., Galitz A., Eickhoff B., Namuth L., Schumacher T., Esmaeili H.R., Wörheide G., Teimori A. (2020). Molecular biodiversity of Iranian shallow water sponges. Syst. Biodivers..

[10] Wilkinson C. (2002).

[11] Raijman-Nagar L., Goren L., Shefer S., Ilan M. (2024). Sponge abundance and diversity patterns in the shallow and mesophotic reefs of the northern Red Sea. Front. Mar. Sci..

[12] Uriz M.J. (2006). Mineral skeletogenesis in sponges. Can. J. Zool..

[13] Meyer C.P., Paulay G. (2005). DNA barcoding: error rates based on comprehensive sampling. PLoS Biol..

[14] Schönberg C.H.L. (2021). No taxonomy needed: sponge functional morphologies inform about environmental conditions. Ecol. Indic..

[15] Łukowiak M., Van Soest R., Klautau M., Pérez T., Pisera A., Tabachnick K. (2022). The terminology of sponge spicules. J. Morphol..

[16] Zea S., Henkel T.P., Pawlik J.R. (2014). The Sponge Guide: a Picture Guide to Caribbean Sponges.

[17] Messing C.G., Díaz M.C., Kohler K.E., Reed J.K., Rützler K., Soest R.W.M., Wulff J., Zea S. (2009). South Florida Sponges. A guide to identification. https://guide.poriferatreeoflife.org/species_class.html.

[18] Hooper J. 'SPONGE guide'. GUIDE to sponge collection and identification (Version August 2000). https://www.academia.edu/34258606/SPONGE_GUIDE_GUIDE_TO_SPONGE_COLLECTION_AND_IDENTIFICATION_Version_August_2000.

[19] Chombard C., Boury-Esnault N., Tillier S. (1998). Reassessment of homology of morphological characters in tetractinellid sponges based on molecular data. Syst. Biol..

[20] Rowan R., Powers D.A. (1992). Ribosomal RNA sequences and the diversity of symbiotic dinoflagellates (zooxanthellae). Proc. Natl. Acad. Sci. U. S. A..

[21] Rowan R., Powers D.A. (1991). Molecular genetic identification of symbiotic dinoflagellates (zooxanthellae). Mar. Ecol. Prog. Ser..

[22] Altschul S.F., Madden T.L., Schäffer A.A., Zhang J., Zhang Z., Miller W., Lipman D.J. (1997). Gapped BLAST and PSI-BLAST: a new generation of protein database search programs. Nucleic Acids Res..

[23] Tamura K., Nei M. (1993). Estimation of the number of nucleotide substitutions in the control region of mitochondrial DNA in humans and chimpanzees. Mol. Biol. Evol..

[24] Kumar S., Stecher G., Li M., Knyaz C., Tamura K. (2018). Mega X: molecular evolutionary genetics analysis across computing platforms. Mol. Biol. Evol..

[25] Hooper J.N.A., van Soest R.W.M., Hooper J.N.A., van Soest R.W.M. (2002). Systema Porifera: a Guide to the Classification of Sponges.

[26] Pulitzer-Finali G., Morton B.S., Tseng C.K. (1982). The Marine Flora and Fauna of Hong Kong and Southern China.

[27] Stuart O.R., Arthur D. (1886). Preliminary report on the monaxonida collected by H.M.S. ‘Challenger. Ann. Mag. Nat. Hist..

[28] Raijman-Nagar L., Shefer S., Feldstein-Farkash T., Novak L., Ilan M. (2023). New Negombata species discovered: latrunculin mystery solved. Coral Reefs.

[29] Hall K.A., Sutcliffe P.R., Hooper J.N.A., Alencar A., Vacelet J., Pisera A., Folcher E., Bourgeois B., Butscher J., Renaud A., Lerouvreur F., Fleurisson D., Orempuller J., Maihota N., Levy P., Hertrich L., Petek S., Debitus C., Petek Sylvain, Debitus Cécile (2017). Sponges of Polynesia.

[30] Kelly-Borges M., Vacelet J. (1995). A revision of Diacarnus Burton and Negombata de Laubenfels (Demospongiae: Latrunculiidae) with descriptions of new species from the west central Pacific and the Red Sea. Memoir. Queensl. Mus..

[31] N. B. Andersen, ‘underwater-image-color-correction’. GitHub. Available online at: https://github.com/nikolajbech/underwater-image-color-correction. Accessed on 14 June 2024.

[32] Patantis G., Rahmadara G., Elfidasari D., Chasanah E. (2013). Molecular identification of sponges obtained from seribu islands national park and their associated bacteria. Squalen Bull. Mar. Fish. Postharvest Biotechnol.

[33] de Paula T.S., Zilberberg C., Hajdu E., Lôbo-Hajdu G. (2012). Morphology and molecules on opposite sides of the diversity gradient: four cryptic species of the Cliona celata (Porifera, Demospongiae) complex in South America revealed by mitochondrial and nuclear markers. Mol. Phylogenet. Evol..

[34] Desqueyroux-Faúndez R., Valentine C., Hooper J.N.A., Van Soest R.W.M. (1936).

[35] Voigt O., Eichmann V., Wörheide G. (2012). First evaluation of mitochondrial DNA as a marker for phylogeographic studies of Calcarea: a case study from *Leucetta chagosensis*. Hydrobiologia.

[36] Duran S., Pascual M., Turon X. (2004). Low levels of genetic variation in mtDNA sequences over the western Mediterranean and Atlantic range of the sponge *Crambe crambe* (Poecilosclerida). Mar. Biol..

[37] Wörheide G. (2006). Low variation in partial cytochrome oxidase subunit I (COI) mitochondrial sequences in the coralline demosponge *Astrosclera willeyana* across the Indo-Pacific. Mar. Biol..

[38] Szitenberg A., Becking I.E., Vargas S., Fernandez J.C., Santodomingo N., Wörheide G., Ilan M., Kelly M., Huchon D. (2013). (Porifera, Demospongiae, Spirophorida) based on three molecular markers. Mol. Phylogenet. Evol..

[39] Yang Q., Franco C.M., Sorokin S.J., Zhang W. (2017). Development of a multilocus-based approach for sponge (phylum Porifera) identification: refinement and limitations. Sci. Rep..

[40] Keller C. (1889). Die Spongienfauna des rothen Meeres (I. Hälfte). Z. Wiss. Zool..

[41] Belinky F., Szitenberg A., Goldfarb I., Feldstein T., Wörheide G., Ilan M., Huchon D. (2012). ALG11 – a new variable DNA marker for sponge phylogeny: comparison of phylogenetic performances with the 18S rDNA and the COI gene. Mol. Phylogenet. Evol..

[42] Rotem M., Kashman Y. (1979). ‘New polyacetylenes from the sponge *Siphonochalina* sp. Tetrahedron Lett..

[43] Lévi C. (1965). Spongiaires récoltes par l'expédition Israélienne dans le sud de la Mer Rouge en 1962. Bull. Sea Fish. Res. Stn Israel.

[44] Burton M. (1926). Sponges. Zoological results of the suez canal expedition. Trans. Zool. Soc. Lond..

[45] Gao L., Song Q., Sang J., Xiao Y., Li Z. (2023). Cytobacillus spongiae sp. nov. isolated from sponge *Diacarnus spinipoculum*. Int. J. Syst. Evol. Microbiol..

[46] El Sayed K.A., Hamann M.T., Hashish N.E., Shier W.T., Kelly M., Khan A.A. (2001). Antimalarial, antiviral, and antitoxoplasmosis norsesterterpene peroxide acids from the Red Sea sponge *Diacarnus erythraeanus*. J. Nat. Prod..

[47] Lefranc F., Nuzzo G., Hamdy N.A., Fakhr I., Moreno Y., Banuls L., Van Goietsenoven G., Villani G., Mathieu V., van Soest R., Kiss R., Ciavatta M.L. (2013). In vitro pharmacological and toxicological effects of norterpene peroxides isolated from the Red Sea sponge Diacarnus erythraeanus on normal and cancer cells. J. Nat. Prod..

[48] Youssef D.T., Yoshida W.Y., Kelly M., Scheuer P.J. (2001). Cytotoxic cyclic norterpene peroxides from a Red Sea sponge *Diacarnus erythraenus*. J. Nat. Prod..

[49] Youssef D.T. (2004). Tasnemoxides A-C, new cytotoxic cyclic norsesterterpene peroxides from the Red Sea sponge *Diacarnus erythraenus*. J. Nat. Prod..

